# SARS-CoV-2 antibodies in adolescents living with perinatally acquired HIV

**DOI:** 10.4102/sajid.v39i1.579

**Published:** 2024-03-26

**Authors:** Lisa Frigati, Leonore Greybe, Shaun Barnabas, Mark Cotton, Helena Rabie

**Affiliations:** 1 Department of Paediatrics and Child Health, Faculty of Medicine and Health Sciences, Stellenbosch University, Cape Town, South Africa

**Keywords:** SARS-CoV2 antibodies, adolescents, adolescent HIV, perinatal HIV, COVID-19, vaccination

## Abstract

Data on children and adolescents with HIV and coronavirus disease 2019 (COVID-19) co-infection are limited. Clinical and antibody data related to COVID-19 infection in adolescents living with perinatally acquired HIV (ALPHIV) and originally enrolled in the Children with HIV Early Antiretroviral Therapy (CHER) study were collected. We present a descriptive analysis of 53 ALPHIV who were tested for anti-SARS-CoV-2 antibodies. Just over half (53%) of the adolescents tested had positive anti-SARS-CoV-2 antibodies with only one participant describing a prior history of possible symptomatic infection.

**Contribution:**

The study contributes to the understanding of SARS-CoV-2 infection and vaccination practices in HIV-positive adolescents.

## Background

Despite initial concerns that those living with HIV may be excessively affected by coronavirus disease 2019 (COVID-19), current evidence suggests that COVID-19 disease in adolescents living with HIV is comparable to those who are HIV negative.^1,2^ There are limited data on children and adolescents living with HIV and COVID-19 co-infection from African settings.^2^

Adolescents living with perinatally acquired HIV (ALPHIV) may be at increased risk of severe COVID-19 disease because of comorbidities such as chronic lung disease or metabolic complications resulting in poor glycaemic control.^3^ Traditional risk factors for COVID-19 include high body mass index, diabetes, chronic lung disease and renal disease. Adolescents living with perinatally acquired HIV have been shown to have a higher burden of many of these conditions.^4^

The aim of this study was to review clinical and antibody data related to COVID-19 in adolescents originally enrolled in the Children with HIV Early Antiretroviral Therapy (CHER) study.

## Methods

The study, based at Tygerberg Hospital’s paediatric infectious diseases clinic and the Family Centre for Research with Ubuntu (FAMCRU) in Cape Town, South Africa, was a cross-sectional study that was nested in a larger study that is exploring cardiovascular risk factors in adolescents who are still in care at Tygerberg Hospital and were previously enrolled in the original CHER study.

Enrolment took place from October 2022 to March 2023. Inclusion criteria were any adolescent between the age of 15 and 20 years, which had perinatally acquired HIV infection and had been on ART for at least 6 months. All those who consented were included. The COVID-19 vaccination became available for adolescents older than 12 years of age in October 2021. History of COVID-19 disease or prior COVID-19 vaccination was self-reported. In addition, a retrospective review of laboratory results was performed to ascertain whether a COVID-19 polymerase chain reaction (PCR) test had been requested for any of the participants.

Evidence of prior COVID-19 infection was based on a positive anti-SARS-CoV-2 nucleocapsid antibody (Abbott) test performed at the National Health Laboratory Service.^5^ The HIV viral load (Alinity^®^ M HIV-1 Test Abbott, Chicago, IL) and CD4 counts (AQUIOS^®^ CL Beckman Coulter, Brea, CA) were also measured. A chi squared test was used to examine the association between anti-SARS-CoV-2 antibodies and HIV viral load. Stellenbosch University Research Ethics Committee approved this study.

### Ethical considerations

The Stellenbosch Health Research Ethics Committee approved this study. Informed consent was taken from caregivers of study participants and participants provided assent. The study was funded by National Institutes of Health (NIH), reference number: 1D43TW010937-01.

## Results

Fifty-three participants, median age 16.5 years (IQR: 16.4–16.8), were enrolled, 29 (54%) were male and 47 (89%) had a viral load of < 50 copies/mL. The median CD4 count was 708 cells/μL (IQR: 536–871). Median time on antiretroviral treatment (ART) was 16.1 years (IQR: 15.8–16.5). Median BMI was 21.6 kg (IQR: 19.5–24.3) and 13 (25%) had a BMI > 25 that is considered overweight. No participant reported smoking cigarettes although 25 (47%) smoked cannabis. Four (8%) participants had documented evidence of chronic lung disease, two (4%) had hepatic steatosis and all had normal glucose at the study visit.

No participants had laboratory documentation of COVID-19 PCR testing prior to study enrolment or a history of hospitalisation since the start of the COVID-19 epidemic. One participant had a history of symptoms suggestive of mild COVID-19 disease, but no participants presented to medical services or were admitted to hospital. Seven (13%) participants reported having received COVID-19 vaccination. Twenty-eight (53%) participants tested positive for anti-SARS-CoV-2 nucleocapsid antibodies. A total of 19 (68%) of those who had positive SARS-CoV-2 antibodies were male (*p* = 0.04).

There was no association between having positive anti-SARS-CoV-2 antibodies and having an unsuppressed HIV viral load (VL > 50 copies/mL). There was also no association between having a BMI > 25, having a CD4 count < 500 cells/μL or having had a previous history of having COVID-19 disease and having postive anti-SARS-CoV-2 antibodies (Table 1).

**TABLE 1:** Participants’ characteristics stratified for antibody status.

Participants’ characteristics	SARS-CoV-2 antibody positive (*N* = 28)		SARS-CoV-2 antibody negative (*N* = 25)		*P* Value
	*n*	%	*n*	%	
VL > 50 copies/mL	5	5	4	16	0.9
CD4 < 500 cells/μL	1	1	1	4	0.9
BMI > 25 kg/m^2^	6	6	7	54	0.6
H x of COVID19 vaccination	4	4	3	12	0.8
H x of COVID-19 disease	1	4	0	0	NA

Ab, antibody; VL, viral load; ml, millilitre; μL, microlitre; IQR, interquartile range; BMI, body mass index; kg/m^2^, kilogram per square meter; Hx, history.

## Discussion

These results suggest that in this cohort of ALPHIV that started ART very early in life, over half of ALPHIV had had prior COVID-19 infection with none having had severe disease.

This is similar to another study performed in Cape Town in which over 80% of 283 ALPHIV had positive serology for COVID-19 infection. In that cohort, the median age of starting ART was higher, but 76% had an undetectable viral load.^6^

In our study population, viral load and CD4 count results suggest that past COVID-19 infection did not result in measurable loss of control of HIV infection or worsening of HIV disease.

This study also highlights the fact that the prevalence of traditional risk factors for severe COVID-19 disease may differ in African populations with no participant reporting smoking cigarettes although many reported using other substances and smoking traditional herbs. We did not assess the association between glucose and positive SARS-CoV-2 antibodies as we just performed a once off measurement of glucose.

In addition, this study demonstrates the poor uptake of COVID-19 vaccination in adolescence despite the safety of vaccination in this population and regular clinic attendance.^7^

## Conclusions

This limited analysis of a small number of ALPHIV on ART suggests that asymptomatic SARS-COV-2 infection is common and that many of these adolescents still need to be vaccinated.
